# A benefit–cost analysis of different response scenarios to COVID‐19: A case study

**DOI:** 10.1002/hsr2.286

**Published:** 2021-06-04

**Authors:** David C. Cook, Rob W. Fraser, Simon J. McKirdy

**Affiliations:** ^1^ School of Agriculture and Environment The University of Western Australia Perth Western Australia Australia; ^2^ Harry Butler Research Institute Murdoch University Perth Western Australia Australia; ^3^ Department of Economics The University of Kent Canterbury UK

**Keywords:** natural disaster, policy making, public policy, simulation modeling

## Abstract

**Background:**

This paper compares the direct benefits to the State of Western Australia from employing a “suppression” policy response to the COVID‐19 pandemic rather than a “herd immunity” approach.

**Methods:**

An S‐I‐R (susceptible‐infectious‐resolved) model is used to estimate the likely benefits of a suppression COVID‐19 response compared to a herd immunity alternative. Direct impacts of the virus are calculated on the basis of sick leave, hospitalizations, and fatalities, while indirect impacts related to response actions are excluded.

**Results:**

Preliminary modeling indicates that approximately 1700 vulnerable person deaths are likely to have been prevented over 1 year from adopting a suppression response rather than a herd immunity response, and approximately 4500 hospitalizations. These benefits are valued at around AUD4.7 billion. If a do nothing policy had been adopted, the number of people in need of hospitalization is likely to have overwhelmed the hospital system within 50 days of the virus being introduced. Maximum hospital capacity is unlikely to be reached in either a suppression policy or a herd immunity policy.

**Conclusion:**

Using early international estimates to represent the negative impact each type of policy response is likely to have on gross state product, results suggest the benefit–cost ratio for the suppression policy is slightly higher than that of the herd immunity policy, but both benefit–cost ratios are less than one.

## 1 INTRODUCTION

Since an outbreak of a novel coronavirus (COVID‐19) was detected in Wuhan, China, in late‐2019, the virus has quickly spread throughout the world. The resultant pandemic has seen governments implementing policies to reduce the morbidity and mortality from acute infections.^1^ While mitigating the spread of the virus, however, these response policies have had large impacts on societies and economies.^1^, ^2^ Despite having to react to the virus quickly, it is still important that governments use traditional policy evaluation tools like benefit–cost analysis to evaluate the net effects of alternative response strategies on their constituents.

The detection of COVID‐19 in Western Australia in February 2020 provides an example of a response by a discrete region where the State government declared a State of Emergency shortly after the first detection, providing police and other services jurisdiction to enforce quarantine and self‐isolation measures to contain spread. This response, termed “suppression,” is consistent with policies implemented by governments around the world aimed at slowing and eventually reversing epidemic growth, reducing case numbers to low levels, and maintaining that situation indefinitely.^3^

As an alternative, the Western Australian government could have chosen to follow a different management strategy based on the principle of “herd immunity.” A herd immunity response policy assumes the likelihood of an infected individual coming into contact with a susceptible individual is lessened with a proportion of the population (but not all) being immune.^4^, ^5^ This effect may soon be achieved through widespread vaccination, but given the disease‐induced herd immunity level for COVID‐19 is relatively low,^6^ it could be achieved by allowing infections and recovery to occur in less‐vulnerable sections of the population. Sweden has officially adopted this natural science herd immunity approach in its COVID‐19 mitigation strategy.^7^

This paper describes an S‐I‐R (susceptible‐infectious‐resolved) model that is used to estimate the likely benefits of a suppressed COVID‐19 response compared to a herd immunity alternative. A 1‐year period from the time of the virus' initial introduction is simulated, and the resultant number of infections, hospitalizations, and deaths is estimated for these two policy scenarios and for a counterfactual “do nothing” scenario in which the virus spreads through the population without concentrated efforts to contain it. The model allows for the possibility of the Western Australian hospital system crashing under the strain of COVID‐19 cases.

Section 2 outlines the model used to generate COVID‐19 infection numbers under different response policies and its parameterization. Section 3 presents model outputs; specifically, the number of cases expected in Western Australia under suppression and herd immunity responses, the direct costs of each policy, the effect of each policy on the State's hospital sector, and the sensitivity of costs to uncertainties in model parameters. Section 4 uses the results and recent anecdotal evidence of response costs to present an indicative benefit–cost analysis. And finally, Section 5 summarizes key findings and draws conclusions.

## 2 METHODS

### 2.1 Model

A simple S‐I‐R model is used to simulate the spread of COVID‐19 through the Western Australia population. Its components are:

(1) $$S_{t}=S_{t-1}-R_{0}\beta I_{t-1}\left(\frac{S_{t-1}}{S_{t_{0}}}\right)$$

(2) $$I_{t}=I_{t-1}+R_{0}\beta I_{t-1}\left(\frac{S_{t-1}}{S_{t_{0}}}\right)-\gamma \left(1-\delta^{I}A\right)I_{t-1}-\gamma \delta^{I}AI_{t-1}$$

(3) $$R_{t}=R_{t-1}+\gamma I_{t-1}$$

(4) $$N=S_{t}+I_{t}+R_{t}$$

In Equations (1), (2), (3), (4), *S*_*t*_ is the number of susceptible individuals within a population *N* in time period *t* after the initial introduction of the virus who have not been infected; *I*_*t*_ is the number of people within the population in period *t* who are infected and can transmit the virus; *R*_*t*_ is the number of resolved cases in period *t* who are no longer capable of transmitting the virus, including those who have returned to health and those who have died; *R*_0_ is the average number of people that one infectious person will go on to infect; *β* is the final outbreak size expressed as a proportion of the total susceptible population; *γ* is the resolution rate of infections; *δ* ^*I*^ is the case fatality rate among people with a high risk of severe infection; and *A* is the proportion of the population at high risk of severe infection.

Equation (1) states that the number of susceptible individuals in the population at time *t* is equal to the number of susceptible people in the previous period minus newly infected individuals. While acknowledging that under certain conditions the size of an epidemic can be predicted by *R*_0_,^8^, ^9^, ^10^ small variations in these conditions can lead to very different‐sized epidemics.^11^ Rather than specifying the complex relationship between *R*_0_ and *β*, the simple model presented here assumes they are independent variables.

Equation (2) states that the number of people capable of transmitting the virus (or infectives) in period *t* is equal to the number of infectives in the previous period plus the number of new infectives minus the number of resolved infections. Resolved infections are those that have resulted in either a return to relative health or death, and as such transmission can no longer occur. Both resolutions are assumed to take the same number of periods to resolve, $\frac{1}{\gamma }$. Note that this specification of Equation (2) assumes nobody outside the group at high risk of severe infection dies from the virus.

Equation (3) states that the number of people who have been infected with the virus and can no longer transmit it (ie, recovered or deceased) in time period *t* is equal to the number of resolved cases in the previous time period plus the number of newly resolved cases.

The number of fatal infections is partially dependent on the ability of the hospital system to cope with the number of COVID‐19 patients. When hospitals reach capacity, patients must be turned away and cared for at other locations (eg, home care, hospices, makeshift triage centers, etc.) where the case fatality rate for those at risk of severe infections is higher than in hospital care. The number of fatal infections *t* days after virus introduction, *D*_*t*_, is determined by the piecewise function:

(5) $$D_{t}=\left\{\begin{array}{c} D_{t-1}+\gamma \delta^{I}{\mathrm{AI}}_{t}\;\text{if}\;{\mathrm{\eta I}}_{t}\leq B_{t}\\ D_{t-1}+\gamma \delta^{I}{\mathrm{AB}}_{t}+\gamma \delta^{E}A\left({\mathrm{\eta I}}_{t}-B_{t}\right)\;\text{if}\;{\mathrm{\eta I}}_{t}>B_{t} \end{array}\right.$$

Here, *D*_*t*_ is number of deceased individuals within a population *N* in time period *t* after the virus is introduced; *η* is the proportion of infected individuals requiring hospitalization; *B*_*t*_ is the number of hospital beds available for COVID‐19 patients on day *t* after virus introduction; and *δ* ^*E*^ is the case fatality rate among people with a high risk of severe infection who are turned away from hospitals when no beds are available (ie, *δ* ^*E*^ > *δ* ^*I*^).

Equation (5) states that if the number of infected individuals requiring hospitalization does not exceed capacity, the number of deaths attributable to COVID‐19 *t* days after the virus is introduced is equal to previous deaths plus the number of new deaths among those at high risk of severe infection. If hospitals reach capacity, new deaths include infected individuals at high risk of severe infection turned away from hospitals who experience higher case fatality rates than similar patients in hospital care.

By simulating values for *I*_*t*_ and *D*_*t*_ from Equations (2) and (4), costs imposed by COVID‐19 can be estimated as it moves through the Western Australia population. The costs related to nonfatal infections *t* days after virus introduction, $C_{t}^{I}$, are:

(6) $$C_{t}^{I}=\left\{\begin{array}{c} I_{t}\left(\mathrm{\eta H}+\mathrm{\omega W}\right)\;\text{if}\;I_{t}\eta \leq B_{t}\\ {\mathrm{\eta HB}}_{t}+\mathrm{\omega W}I_{t}+E\left(I_{t}\eta -B_{t}\right)\;\text{if}\;I_{t}\eta >B_{t} \end{array}\right.$$

Here, *H* is the cost of that hospitalization; *ω* is the proportion of infected individuals in need of sick leave; *W* is the average fortnightly wage rate (ie, assuming two working weeks are lost as a result of illness); and *E* is the extra costs society pays to treat those turned away from hospitals in other locations.

Equation (6) states that if the number of cases is less than the number of available hospital beds, the costs related to nonfatal infections will depend on the number of infected individuals requiring hospitalization, the cost of hospitalization, the number of people who require time off work to recover from the virus, and the average fortnightly wage. If the number of cases is greater than the capacity of the State's hospitals, the costs related to nonfatal infections depend on the maximum number of beds and the cost of hospitalization, the number of people who require time off work to recover from the virus, and the average fortnightly wage, plus the societal costs involved in providing care for those patients turned away from hospitals.

The cost of fatal infections at time *t* days after the introduction of the virus, $C_{t}^{D}$ is calculated as:

(7) $$C_{t}^{D}=D_{t}L\;$$

Here, *D*_*t*_ is the number of fatal infections occurring *t* days after the virus is introduced to the population; and *L* is the *value of a statistical life*—a measure of the willingness of individuals to pay for a reduction in mortality risk sufficient to lower the expected number of fatalities by one over a given period of time.

Equation (7) states that the cost of fatal infection costs will be determined by the number of fatal infections and the statistical value of lives lost.

With simulated values for $C_{t}^{I}$ and $C_{t}^{D}$, the combined total nonfatal and fatal infection costs of the virus (*C* ^*T*^) over *n* days are:

(8) $$C^{T}=\sum_{t=1}^{n}{C_{t}^{I}+C}_{t}^{D}$$

In the results section, $C_{t}^{I}$, $C_{t}^{D},$ and *C* ^*T*^ are reported for: (a) the counterfactual “do nothing” policy in which the virus is permitted to spread throughout the Western Australian population without special measures to slow infection, (b) the suppression policy reflecting the policy currently in place, and (c) a herd immunity scenario in which the virus is allowed to spread through the nonvulnerable portion of the population while vulnerable portion is protected through isolation.

Specifically, the do nothing scenario involves no restrictions to people movements or behavior being enforced by the Western Australian government. This does not mean people will not take personal decisions to minimize risks associated with virus spread, such as social distancing, self‐isolating when ill, or wearing face masks in public. With the widespread media coverage of the spread and impact of COVID‐19, particularly in other parts of the world, it is reasonable to expect Western Australians to adopt these measures regardless. However, in the do nothing scenario, they are not made mandatory.

The suppression scenario involves a State of Emergency declaration providing the Western Australian police force and Chief Health Officer with the jurisdiction to enforce quarantine and self‐isolation measures consistent with the national response management approach. This includes the closure of schools, daycare centers, and nonessential businesses. Anyone arriving into the State from overseas or interstate is required to self‐isolate for 14 days, and strict border controls for road, rail, air, and sea entry points are in place. Nonessential indoor gatherings of >100 people are prohibited, and a “one person per four square metres of floor space” applies. It is assumed that these measures have the effect of lowering the transmission of the COVID‐19 virus, reflected in a lower *R*_0_ value and lower *A* parameter when compared to the do nothing approach.

The herd immunity response entails the isolation and protection of susceptible members of the population, while the virus is permitted to spread unabated through the nonvulnerable population. Schools and businesses would remain open, and the borders open to interstate and international passengers. Restrictions would apply to the movement of and contacts with vulnerable members of the population, including the elderly, people with pre‐existing medical conditions, and newborns. It is assumed that these measures would result in *R*_0_ and *A* values that are lower than those expected in the do nothing scenario, but higher than those expected under the suppression policy scenario. Note that full details of the specification of parameter values are provided in Section 2.2.

The total benefit achieved by pursuing either the suppression policy ($B_{S}^{T}$) or herd immunity policy ($B_{H}^{T}$) over *n* days is measured by avoided costs and, therefore, is calculated as the difference in total costs incurred in these scenarios and in the do nothing scenario:

(9) $$B_{S}^{T}=\sum_{t=1}^{n}{C_{D}^{T}-C}_{S}^{T}$$

(10) $$B_{H}^{T}=\sum_{t=1}^{n}{C_{D}^{T}-C}_{H}^{T}$$

Here, $C_{D}^{T}$, $C_{S}^{T}$, and $C_{H}^{T}$ are the total nonfatal and fatal infection costs likely to occur over *n* days under do nothing, suppression, and herd immunity response policies, respectively.

Based on the parameter values specified in Section 2.2, the virus simulation is run for 365 days following an initial introduction. Using the Monte Carlo method, 10 000 iterations of the input model defined in this section are run to generate probability distributions of possible outcomes. The results reveal all possible events that could happen according to the model's structure and parameters, and the probability of each outcome occurring.

### 2.2 Parameters

Model parameters and their assumed values, drawn from the relevant literature, appear in Table 1 and are discussed below. Pert distributions are preferred when evidence and expert opinions on parameter values are mixed,^12^ and uniform distributions are used to represent highly uncertain parameters.

**TABLE 1:** Parameters of the model

Parameter	Description	Do nothing	Suppression	Herd immunity
*N*	Number of susceptible people at time 0	2.6 million	2.6 million	2.6 million
$I_{t_{0}}$	Number of infected people at time 0	1	1	1
*R*_0_	Average number of infections transmitted by one infective	Pert(1.5,2.5,3.5)	Pert(0.3,1.319,2.607)	Pert(1.275,2.25,3.325)
*β*	Size of overall epidemic as a proportion of total population	Pert(0.25,0.3,0.35)	Pert(0.25,0.3,0.35)	Pert(0.25,0.3,0.35)
*γ*	Resolution rate	Pert(0.3,0.325,0.35)	Pert(0.3,0.325,0.35)	Pert(0.33,0.358,0.385)
*η*	Rate of infective hospitalization	Pert(0.01,0.015,0.02)	Pert(0.008,0.012,0.016)	Pert(0.009,0.014,0.018)
*H*	Cost of hospitalization	Pert($13 800,$23 000,$41 400)	Pert($13 800,$23 000,$41 400)	Pert($13 800,$23 000,$41 400)
*B*	Maximum number of hospital beds available	Pert(5670,6630,7460)	Pert(5670,6630,7460)	Pert(5670,6630,7460)
*ω*	Proportion of infectives requiring time off work	Uniform (0.03,0.13)	Uniform (0.03,0.13)	Uniform(0.03,0.13)
*W*	Wage costs	$2660/fortnight	$2660/fortnight	$2660/fortnight
*E*	Cost of treating excess patients turned away from hospitals	$9200/day	$9200/day	$9200/day
*A*	Proportion of population at high risk of severe infection	Pert(0.05,0.1,0.15)	Pert(0.005,0.0175,0.0375)	Pert(0.0125,0.0375,0.075)
*δ* ^*I*^	Case fatality rate	Pert(0.007,0.01,0.014)	Pert(0.007,0.01,0.014)	Pert(0.006,0.009,0.013)
*δ* ^*E*^	Case fatality rate for patients turned away from hospitals	Pert(0.008,0.0125,0.045)	Pert(0.008,0.0125,0.045)	Pert(0.007,0.0112,0.0405)
*L*	Age‐adjusted value of a statistical life	Uniform($280 000,$565 000)	Uniform($280 000,$565 000)	Uniform($280 000,$565 000)

The total population of Western Australia is currently 2 590 290.^13^ This value is used for the parameter *N*.

Preliminary estimates of the *R*_0_ for COVID‐19 indicate a broad range of values between 1.4 and 4.0.^11^, ^14^, ^15^, ^16^ It is specified in the model using the pert distribution pert(1.5,2.5,3.5) under the do nothing scenario. Following Ferguson et al,^3^ it is assumed actions taken in the suppression policy option (ie, social distancing, case isolation, household quarantine, and school and university closures) will reduce *R*_0_ to close to one, although a range of possibilities is considered. *R*_0_ is assumed to change by uniform (−75%,−30%) under the suppression policy and by uniform(−15%,−5%) under the herd immunity policy.

The final outbreak size expressed as a proportion of the total susceptible population, *β*, is estimated to be between 5% and 40%.^11^ This wide range reflects differing accounts of its breadth in different countries. It is specified in the model using a narrower distribution, pert(0.25,0.3,0.35).

The resolution rate, *γ*, is the inverse of the infectious period for an average person. Early indications are that the *γ* is around 33%.^17^ It is specified in the do nothing scenario as pert(0.3,0.325,0.35). Under the herd immunity policy, the average infected person is likely to be of a younger age than in either the do nothing or suppression scenarios. Therefore, a higher resolution rate of pert(0.33,0.358,0.385) is assumed, representing a 10% increase.

Similarly, the case fatality rate, *δ* ^*I*^, is likely to fall as the composition of infectives changes according to the scenario. For the population as a whole, it is specified as pert(0.007,0.01,0.014)^18^, ^19^, ^20^ and is assumed constant over the do nothing and suppression scenarios. As the average age of infectives is younger in the herd immunity scenario, the case fatality rate is reduced by 10% to pert(0.006,0.009,0.013). The importance of this specification is discussed in the sensitivity analysis in Section 3.4.

**FIGURE 1:**
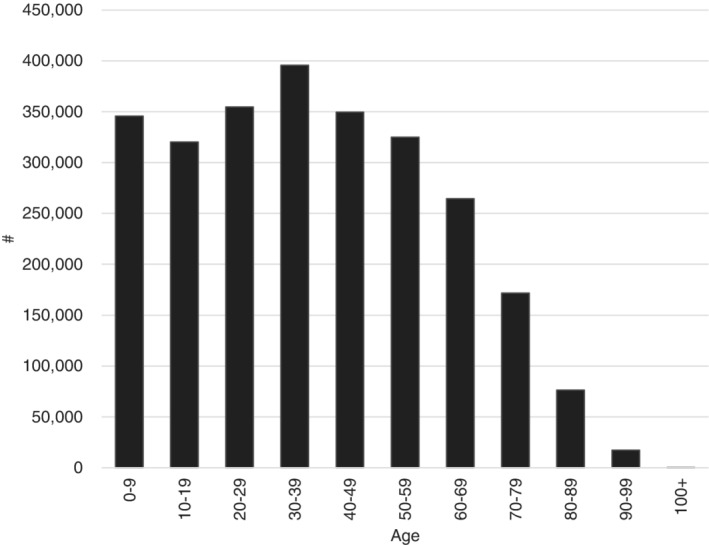
Age of the Western Australian population, 2019.^13^ People of age 70+ are deemed to be at the highest risk of fatal infection, representing 10% of the total population

The proportion of the population at greatest risk, *A*, is approximated using age demographic data for Western Australia.^13^ This is shown in Figure 1, below. Assuming people between the ages of 70 and 100+ to be at the highest risk,^3^, ^21^ this accounts for approximately 10% of the Western Australian population. Allowing variability around this mean value, *A* is specified in the model as pert (5%,10%,15%). Using Ferguson et al^3^ as a broad indication of social distancing effects, its parameter value is changed by uniform (−90%,−75%) and uniform (−75%,−50%) under the suppression and herd immunity policies, respectively.

Nguyen‐Van‐Tam et al^22^ estimated the proportion of infections requiring hospitalization (*η*) for the 2009 H1N1 influenza pandemic at around 1%. Supposing the hospitalization rate of COVID‐19 infections is at least this high, it is assumed to be between 1.0% and 2.2% [ie, pert(0.01,0.016,0.022)] in the do nothing scenario. This is assumed to change by −20% under the suppression policy [ie, Pert(0.008,0.012,0.016] and by −10% under the herd immunity policy [ie, Pert(0.009,0.014,0.018)].

The cost of hospitalization, *H*, is specified as $4600 per day in today's dollars,^23^ with an average length of hospital stay of 3 to 9 days with a most likely duration of 5 days.^24^

The proportion of infections requiring time off work, *ω*, is assumed to be between 3% and 13% [ie, uniform(0.03,0.13)]. This distribution is estimated on the basis that approximately 80% of people infected with COVID‐19 show mild symptoms, while 20% exhibit more severe symptoms,^21^, ^25^ and around 64% of people infected are likely to be of working age.^13^ When infected workers do stay at home, it is assumed they are absent for two full weeks of work. Hence, the average fortnightly wage for Western Australian workers is used to approximate the parameter *W*. A value of $2660/fortnight is used.^26^ Given the majority of 70+ year olds are retired from the workforce, this parameter is assumed constant across the three scenarios.

It is difficult to estimate the cost society incurs as a result of additional care for excess patients turned away from hospitals when capacity is reached, *E*. These patients require adequate nursing availability, 24‐hour on‐call medical advice and home support, patient‐centered planning, daily nursing review and adjustment of individual care plan, professional multidisciplinary team support (eg, occupational therapy, physiotherapy, social work), and a discharge hand‐over to ongoing support services.^27^, ^28^ Without economies of scale, the marginal cost of these services tends to be higher in makeshift facilities or homes than in hospitals, and the duration of health episodes can be longer.^29^ The cost of providing services to infected patients turned away hospitals is assumed to be double the hospitalization costs, *H*. ^28^

The case fatality rate of patients turned away from hospitals, *δ* ^*E*^, is also difficult to estimate. Various studies have shown a negligible difference in treatment outcome between patients utilizing home hospital care and those treated in hospitals.^28^, ^30^, ^31^, ^32^, ^33^ Notably, Vianello et al^34^ also found no statistical difference in treatment failure rate for patients treated for respiratory tract infections at home or in hospital. However, no COVID‐19‐specific estimates are available. In the absence of empirical evidence and to allow for a range of possibilities, it is assumed *δ* ^*E*^ > *δ* ^*I*^ by a factor of uniform (0%,50%).

The age‐adjusted value of a statistical life, *L*, represents the marginal rate of substitution between wealth and mortality risk corrected for the age of the population studied.^35^ In a review of empirical estimates relevant to Australia, Abelson^36^ recommended a value of a statistical life of $3.5 million, which was later revised to $4.2 million,^37^ or $4.7 million today's dollars. If everyone faced the same risk of fatal COVID‐19 infection and statistical life value was identical, this figure could simply be multiplied by the number of COVID‐19 deaths prevented over time in each policy scenario to give the total value of lives saved.^38^ However, those at risk of fatal COVID‐19 infection are disproportionately elderly.^39^, ^40^, ^41^ The parameter *L* in the current study was estimated by dividing the value of a statistical life by the average remaining life expectancy for the population and then multiplying through by the expected years of life extension attributable to each policy.^42^ So, given the average life expectancy in Western Australia is 83.2 years,^43^ and avoidance of COVID‐19 infection has an expected life extension of 5‐10 years, *L* is $0.3‐0.6 million.

In view of the uncertainty of the assumed parameter values in this analysis, the results in the following section are presented first on the basis of the initial specification outlined in this section and subsequently are subjected to a sensitivity analysis.

## 3 RESULTS

### 3.1 Spread

**FIGURE 2:**
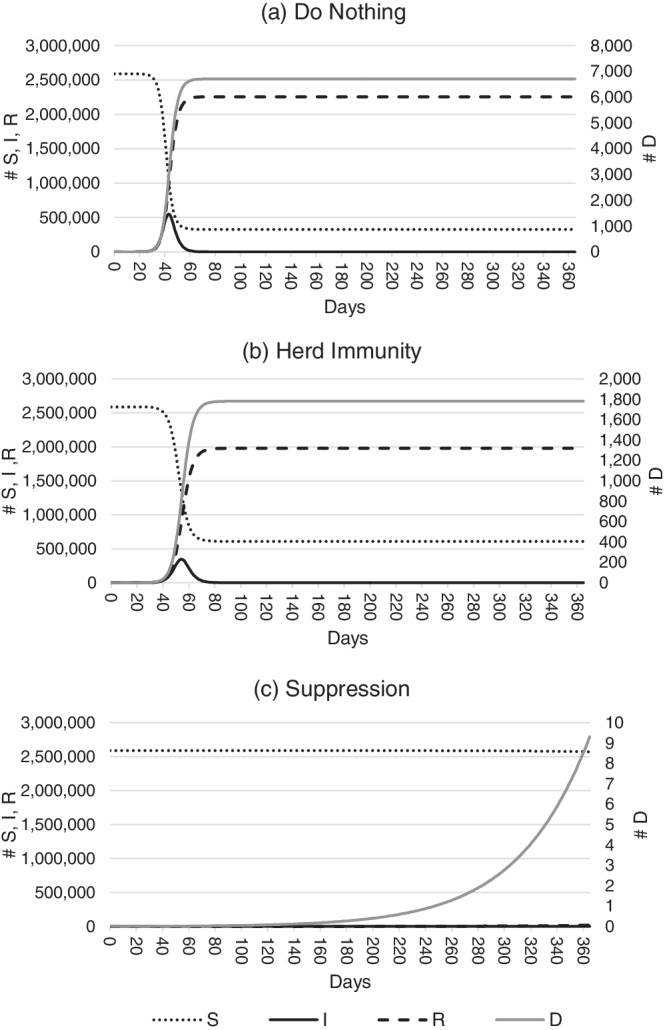
Predicted COVID‐19 infection in Western Australia over 365 days. Panels A‐C show the number of susceptibles, infectives, and resolved cases over time in the do nothing, herd immunity, and suppression scenarios, respectively

Figure 2 shows the number of people affected by the COVID‐19 virus as it spreads through the Western Australian population. Panels (A), (B), and (C) depict the do nothing, herd immunity, and suppression scenarios, respectively, with expected values for *S*, *I*, and *R* shown over 365 days following initial introduction. To produce this figure, parameters in the model are held at their mean values.

Panels (A) and (B) show that the numbers of nonfatal infections are expected to peak at approximately 550 000 cases and 450 000 cases 40‐60 days after the virus' introduction under the do nothing and herd immunity scenarios, respectively. Infections return to near‐zero cases 60‐70 days after introduction. This compares to approximately 1 000 infections in the suppression scenario over the whole year, although this is difficult to see in panel (C) due to scaling.

Also difficult to see in the figure is the number of fatal infections expected, which reach a maximum of around 7 000 under the do nothing scenario 60‐70 days after the virus' introduction. Under the herd immunity scenario, deaths are expected to reach a maximum of approximately 1 700 over the same period, while nine fatalities are predicted in the suppression scenario. This closely matches observations, with the State having recorded nine deaths from 910 cases as of February 2021 (Department of Health Western Australia, 2021).

### 3.2 Benefits

**FIGURE 3:**
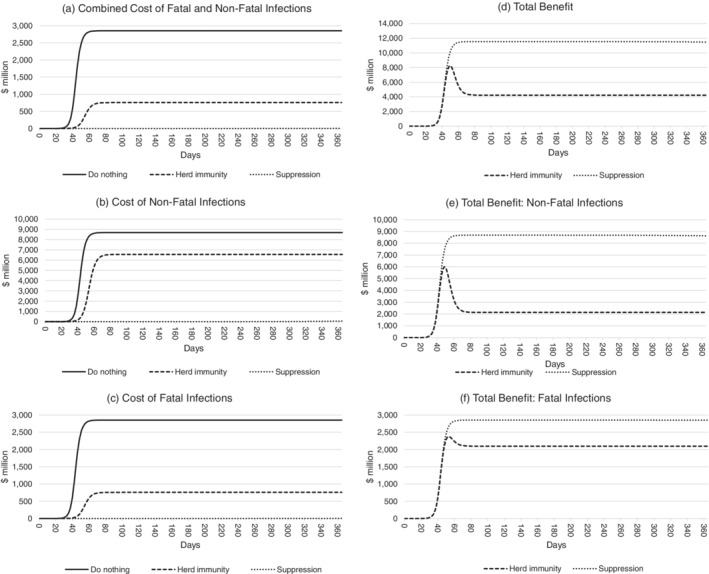
Estimated total benefits of COVID‐19 response policies in Western Australia over 365 days. For each policy, the combined fatal and nonfatal infection costs are shown in panel A, nonfatal infection costs in panel B, and fatal infection costs in panel C. The combined total benefits of the herd immunity and suppression scenarios relative to do nothing are shown in panel D. The value of avoided nonfatal infections is shown in panel E, and the value of avoided fatal infections is shown in panel F

Figure 3 compares the nonfatal infection costs, fatal infection costs, and total costs anticipated under do nothing, herd immunity, and suppression policy scenarios (left panels [A], [B], and [C]) and on this basis estimates the total benefits in terms of avoided costs of the herd immunity and suppression scenarios relative to do nothing (right panels [D], [E], and [F]). Once again, parameter values are held at their mean values to create this figure.

Using the age‐adjusted value of a statistical life measure and average parameter values, panel (C) shows that the costs related to COVID‐19 fatalities are expected to reach a maximum of $2.8 billion 40‐60 days after its introduction under a do nothing scenario. This is reduced to approximately $0.7 billion after 60‐80 days under a herd immunity policy, and approximately $3.8 million after 230‐240 days under a suppression policy. The total benefits in terms of avoided fatal infections costs at the end of the 365 days, shown in panel (F), are approximately $2.1 billion for the herd immunity policy and $2.9 billion for the suppression policy.

Panel (B) shows the costs of nonfatal infections under each scenario. Under a do nothing policy, costs related to nonfatal infections are expected to reach a maximum of just over $8.7 billion 40‐60 days after introduction. A herd immunity policy reduces these costs to a maximum of $6.5 billion 60‐80 days after introduction, while a suppression policy reduces nonfatal infection costs to a maximum of $60.0 million 340‐360 days after introduction. The total benefits related to nonfatal infections after 1 year, shown in panel (E), are approximately $2.1 billion for the herd immunity policy and $8.6 billion for the suppression policy.

Panel (D) indicates the combined nonfatal infection benefits and fatal infection benefits in terms of avoided costs for the herd immunity and suppression scenarios relative to the do nothing scenario. Total benefits are negligible for around 30 days after COVID‐19 introduction. They then spike to a peak of around $11.5 billion in the suppression scenario and $8.2 billion in the herd immunity scenario after 40‐60 days, coinciding with the sudden rise in cases in the do nothing scenario. Total benefits of heard immunity return to a steady state of $4.2 billion 60‐80 days after COVID‐19 introduction.

### 3.3 Hospitalizations

The number of infections requiring hospitalization is expected to strain the Western Australian health sector, and in the case of the do nothing scenario may overwhelm it. Figures released in 2019 indicate the combined number of beds in Western Australian public and private hospitals is 9 949.^44^ Assuming that approximately one‐third of these beds will be occupied by patients with other needs, this leaves around 6 600 beds available at any one time for COVID‐19 patients. If the number of infected individuals requiring hospitalization exceeds this capacity, excess patients will be unable to access appropriate care. As stated in Equation (6), the model assumes these cases have a higher likelihood of resulting in death.

**FIGURE 4:**
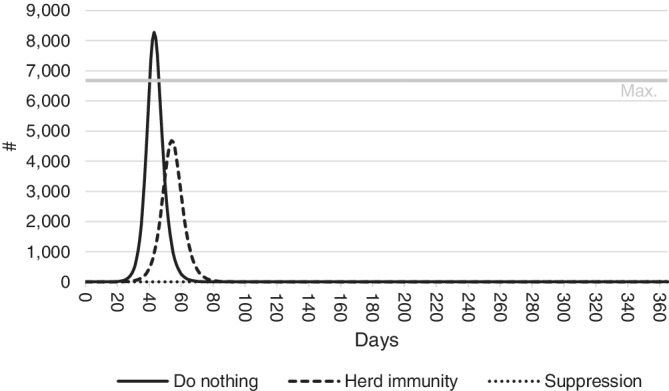
Predicted number of hospitalizations under do nothing, herd immunity and suppression response policies. Patient numbers are expected to exceed the maximum capacity of Western Australian hospitals in the do nothing scenario, but not in either the herd immunity or suppression scenarios

Figure 4 shows the number of people expected to present to the State's public and private hospitals over 365 days, and compares this to the estimated number of hospital beds available. As before, parameters are set to their mean values to produce the figure. Maximum hospital capacity is not expected to be reached under a herd immunity or suppression policy, but under a do nothing scenario, the number of people in need of hospitalization is expected to surpass the number of hospital beds 40‐50 days after COVID‐19 is introduced to the population.

The number of patients turned away when hospitals are at full capacity under a do nothing policy is estimated to be 1 600. Referring back to Figure 3, the effects of the health system crashing can be seen by the rapid escalation of costs in panels (A)‐(C). As capacity is reached, patients turned away result in higher patient care costs and risk of fatalities, causing a rapid escalation of costs under the do nothing scenario.

### 3.4 Sensitivity analysis

**FIGURE 5:**
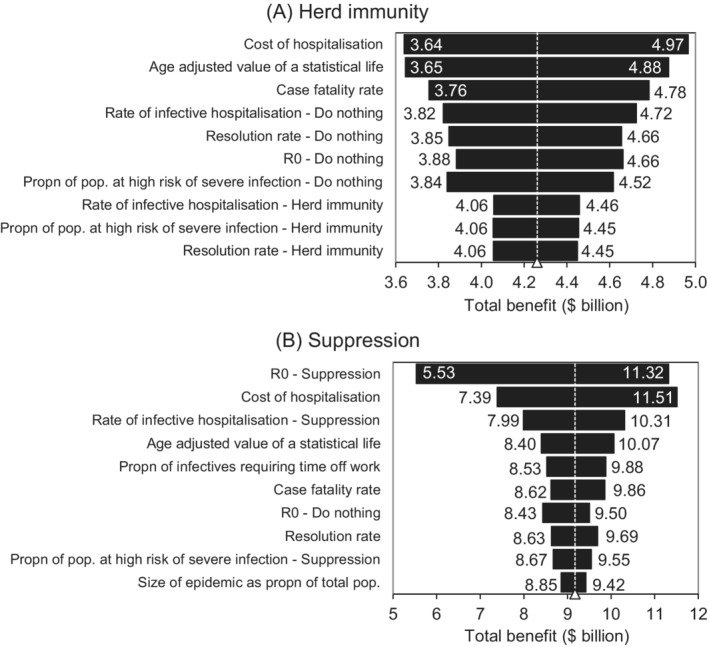
Sensitivity analysis. The length of the bars corresponding to each parameter indicates how responsive the total benefit is to changes in respective parameters. Panel A shows parameter sensitivities for the herd immunity scenario, and panel B parameter sensitivities in the suppression scenario

To determine the effect of uncertain parameter values on model output, each parameter is sampled across its specified range while holding all other parameters constant in Figure 5. Here, the top 10 parameters producing the most change are ranked from top to bottom according to their strength of influence on the total benefit (*B* ^*T*^) in each response scenario. Panel (A) shows sensitivity results for the herd immunity policy (ie, $B_{H}^{T}$, with a mean value of $4.3 billion) and panel (B) for the suppression policy (ie, $B_{S}^{T}$, with a mean value of $9.2 billion).

In panels (A) and (B), results are highly sensitive to changes in the cost of hospitalization. The length of time COVID‐19 patients stay in hospital and subsequent costs depend on their symptoms, which vary in severity. Hospital care can vary from general ward–based care to intensive care where patients may be intubated for mechanical ventilation.^24^, ^45^ Hospitalization cost has a positive relationship with total benefits, and because the input distribution is right‐skewed, the right‐hand‐side sensitivity bar is longer than the left. Changing hospitalization costs from $23 000 in the base case to $13 800 (a change of −40%) lowers total benefit from $4.3 billion to $3.6 billion (−16%) in the herd immunity scenario and from $9.2 billion to $5.5 billion (−40%) in the suppression scenario. Conversely, increasing its value to $41 400 (80%) increases the total benefit to $5.0 billion (16%) in the herd immunity scenario and $11.3 billion (23%) in the suppression scenario.

The age‐adjusted value of a statistical life is also a highly sensitive parameter in both scenarios. Lowering its value from the mid‐point value of $422 500 to $280 000 (a change of −34%) changes total benefits of the herd immunity policy by −16% (to $3.6 billion) and by −9% in the suppression scenario (to $8.4 billion). Likewise, increasing it to $565 000 (a change of +34%) increases the total benefit by approximately 14% (to $4.9 billion) in the herd immunity scenario and by 10% (to $10.1 billion) in the suppression scenario.

In view of its sensitivity, problems with the age‐adjusted value of a statistical life used in this study need to be recognized. Its derivation assumes a linear relationship between age and the value of a statistical life, but in practice, this is highly uncertain.^42^ Evidence suggests the relationship may in fact follow lifetime consumption patterns, being low early and late in life and high in the middle.^46^ Moreover, quality of life is not captured in the age‐adjusted value of a statistical life, meaning the value of life years spent in discomfort due to poor health is considered the same as those spent in good health. However, methods that apply a discount to years of ill‐health or disability, termed “quality adjusted life years,” are problematic, particularly in terms fairness. Negative social perceptions of medical conditions inflate the perceived social benefit of interventions to address them, unfairly raising the value of quality adjusted life years for worse conditions.^47^ Similarly, the more treatable a condition, the higher the perceived social benefit of sufferers receiving treatment before those suffering from less‐treatable conditions.^48^

Figure 5 also shows the results to be sensitive to the rate of infective hospitalization, with changes in the value of this parameter positively related to the level of benefits in both scenarios. In addition, note from Figure 5 that the benefit results are not particularly sensitive to the specification of fatalities outlined in Section 2—with the proportion of the population at high risk of severe infection near the bottom of both panels (A) and (B). However, the case fatality rate (*δ* ^*I*^) is ranked higher in panel (A) than in panel (B) because of the higher number of infectives in the herd immunity scenario, while the case fatality rate for patients turned away from hospitals (*δ* ^*E*^) ranks outside the top 10 parameter sensitivities so does not appear in Figure 5. This is because the number of hospitalizations remains below hospital capacity in both the herd immunity and suppression scenarios (Figure 4). The issue of sensitivity of the results to the specification of the model is further considered in Section 4.

## 4 DISCUSSION OF BENEFITS RELATIVE TO COSTS

To put the total benefits of each policy into perspective, they can be represented as a proportion of the Gross State Product (GSP) of the Western Australian economy. This is equivalent to the national Gross Domestic Product (GDP) but at a State level, capturing income accruing to all individuals and businesses in the economy. Moreover, on this basis, a break‐even economic cost for the policy in terms of its negative impact on GSP can be estimated—thereby indicating the societal cost of implementing a policy that will exactly offset the benefits it is likely to generate. The break‐even policy cost can be estimated as follows:

(11) $$P_{S}=\left(\frac{B_{S}^{T}}{\mathrm{GSP}}\right)\times 100$$

(12) $$P_{H}=\left(\frac{B_{H}^{T}}{\mathrm{GSP}}\right)\times 100$$

Here, *P*_*S*_ and *P*_*H*_ are the break‐even economic costs for suppression and herd immunity policies expressed as a percentage of GSP.

**FIGURE 6:**
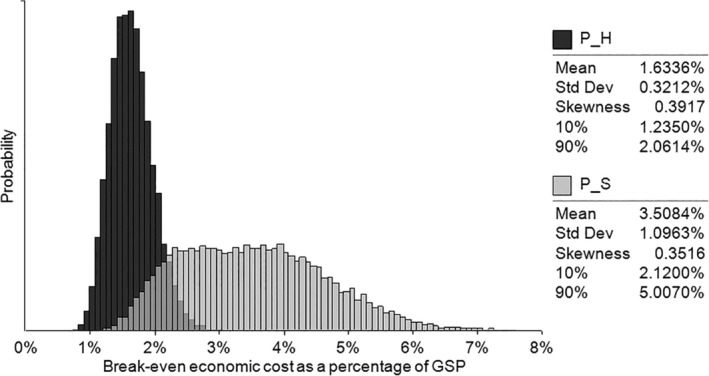
Break‐even economic costs of implementing response policies. The total benefit of each response policy is shown as a proportion of the Gross State Product (GSP) of the Western Australian economy

Given the 2018/19 GSP of Western Australia was $260.6 billion,^49^ histograms of *P*_*H*_ and *P*_*S*_ are show in Figure 6. Results indicate mean *P*_*H*_ is approximately 1.6% of GSP over the 365‐day period simulated in the model (ie, $4.3 billion), whereas estimated mean *P*_*S*_ is 3.5% of GSP (ie, $9.2 billion).

It is too early to have rigorous estimates of the negative economic impact of both types of policy response in terms of decline in GSP with which to compare these estimates of benefits. However, early estimates of the impact of Sweden's herd immunity approach to virus response suggest an overall negative GDP impact for 2020 of between 4% and 6.7% of Sweden's GDP.^50^, ^51^ Early estimates of the broadly suppression type of response for the EU as a whole suggest an overall negative GDP impact for 2020 of between 5% and 12% of EU GDP.^52^

The mid‐points of each of these sets of estimates put the economic cost of Sweden's herd immunity approach to virus response at 5.4% of Sweden's GDP and the economic cost of the EU's suppression approach at 8.5% of the EU's GDP. Using these estimates to illustrate the economic cost of the two policy responses on Western Australia's GSP suggests that the estimated mid‐point benefits of the herd immunity response are greater than the mid‐point costs, and the mid‐point benefits of the suppression policy are just less than the mid‐point costs:

1. Herd immunity: Mid‐point Benefits = 1.6% < Mid‐point costs = 5.4%
2. Suppression: Mid‐point benefits = 3.5% < Mid‐point costs = 8.5%

On this basis, both policy responses are likely to result in a substantial net loss. The herd immunity policy response represents an estimated (mid‐point) benefit–cost ratio (BCR) of 0.3, while suppression represents a (mid‐point) BCR of 0.4.

However, as always, there is considerable uncertainty involved in creating such estimates, and so given the closeness of the estimated BCRs for the two policy responses and that they are both <1, no suggestion as yet exists that one policy response is superior to the other. Similarly, if new information reveals, for example, higher hospitalization costs for the virus than specified here, then the BCRs of both policy responses would be increased.

However, wider impacts of each policy on Western Australian society have been omitted from this assessment that could lower the BCRs substantially. This is particularly true of the suppression option where lockdown periods in Australia have been linked to increased health risks associated with diet and exercise,^53^ heart attack,^54^ mental health,^55^ alcohol abuse,^56^ and domestic violence.^57^ Then again, there may also be additional benefits omitted that would serve to increase the BCR. For example, incidences of violent crime and resultant physical injuries may be reduced under lockdown conditions ordered under the suppression policy.^58^ More research is needed on these broader societal impacts of response actions before a comprehensive policy assessment can be made.

## 5 CONCLUSION

This analysis relies on an S‐I‐R model to predict the likely spread of COVID‐19 infections through the Western Australian population over a period of 365 days under different response policies. Results suggest the societal benefit achieved by a herd immunity response would be approximately $4.3 billion in terms of prevented fatalities, hospitalizations, and sick leave. This is equivalent to 1.6% of total 2018/19 GSP for the state. In comparison, the estimated benefit of a suppression policy is approximately $9.2 billion, equivalent to 3.5% of GSP. It follows that the value of reducing the number of infections under a suppression policy as opposed to herd immunity policy is approximately $4.9 billion. Reduced fatalities amount to a saving of approximately $0.6 billion, while sick leave and hospitalization cost reductions are estimated to be $4.3 billion.

Under a do nothing scenario, the number of people in need of hospitalization is expected to surpass the number of hospital beds 40‐50 days after COVID‐19 is introduced to the population. This is unlikely to happen under a herd immunity or suppression policy. In total, COVID‐19 is estimated to claim the lives of over 7 000 people in Western Australia under a do nothing scenario. Around 75 of these result from hospitals being overwhelmed by the number of people in need of treatment and having to be turned away due to a lack of available hospital beds. The number of fatalities under a herd immunity policy is likely to be around 1 700, while only nine fatalities are predicted under a suppression scenario.

Finally, using preliminary international estimates of the economic costs of each type of policy response on GSP, results suggest that both the suppression and herd immunity policies will fail to generate sufficient benefits to offset the costs of implementing each policy. Net returns may be even smaller if other mental and physical costs of response policies are incorporated into the assessment.

## CONFLICT OF INTEREST

Rob W. Fraser and Simon J. McKirdy certify that they have no affiliations with or involvement in any organization or entity with any financial interest or nonfinancial interest in the subject matter or materials discussed in this manuscript. David C. Cook declares that he has an affiliation with the Western Australian Department of Primary Industries and Regional Development.

## AUTHOR CONTRIBUTIONS

Conceptualization: David C. Cook, Rob W. Fraser, and Simon J. McKirdy

Formal Analysis: David C. Cook

Methodology: David C. Cook, Rob W. Fraser

Writing – Original Draft Preparation: David C. Cook, Rob W. Fraser, and Simon J. McKirdy

Writing – Review & Editing: David C. Cook, Rob W. Fraser, and Simon J. McKirdy

  David C. Cook had full access to all of the data in this study and takes complete responsibility for the integrity of the data and the accuracy of the data analysis.

## TRANSPARENCY STATEMENT

The authors confirm that the manuscript is an honest, accurate, and transparent account of the study being reported; that no important aspects of the study have been omitted; and that any discrepancies from the study as planned (and, if relevant, registered) have been explained.

## FUNDING INFORMATION

The authors received no funding support for research or authorship of this article. Publication costs were supported by the Harry Butler Institute, Murdoch University.

## Data Availability

Data available on request from the authors.
